# The Impact of Chinese Martial Arts Sanda Training on Cognitive Control and ERP: An EEG Sensors Study

**DOI:** 10.3390/s25195996

**Published:** 2025-09-29

**Authors:** Yanan Li, Haojie Li, Haidong Jiang

**Affiliations:** 1 School of Wushu, Shanghai University of Sport, Shanghai 200438, China; liyanan@sus.edu.cn; 2 School of Exercise and Health, Shanghai University of Sport, Shanghai 200438, China; 202121070037@mail.bnu.edu.cn

**Keywords:** sanda sports, cognitive control, electroencephalography (EEG), reaction time, event-related potentials (ERPs), N200, P300, N400

## Abstract

**Objective:** This study aimed to explore the impact of sanda sports experience on cognitive control using electroencephalography (EEG). **Methods:** The study involved 38 male participants, including 19 sanda athletes with over 5 years of training and 19 ordinary college students. A 2 × 4 mixed experimental design was used, with group (sanda athletes vs. ordinary college students) as the between-subjects variable and four experimental conditions (consistent in the previous and current trials, consistent in the previous but inconsistent in the current trials, inconsistent in the previous but consistent in the current trials, and inconsistent in both previous and current trials) as the within-subjects variable. The classic color-word Stroop task was employed to measure cognitive control function through reaction time, accuracy, and event-related potential (ERP) amplitude. **Results:** Sanda athletes exhibited significantly shorter reaction times than ordinary college students across all conditions (*p* < 0.05). There was no significant difference in accuracy between the two groups (*p* > 0.05). ERP results showed that sanda athletes had significantly larger amplitudes for the N200 and P300 components in incongruent trials compared to congruent trials (*p* < 0.05), and significantly larger N400 amplitudes in incongruent trials than ordinary college students (*p* < 0.05). **Conclusions:** Sanda athletes demonstrated faster response speed and enhanced cognitive control abilities, as indicated by ERP components, without sacrificing task accuracy.

## 1. Introduction

Cognitive control, as a core component of executive function, refers to the higher-order neuropsychological process by which individuals dynamically regulate cognitive resources to achieve goal-directed behavior. This ability primarily relies on the prefrontal cortex and its functional networks with other brain regions such as the basal ganglia and parietal cortex [1,2,3]. Existing neuroimaging studies consistently indicate that the dorsolateral prefrontal cortex plays a key role in attention control, the anterior cingulate cortex is specifically responsible for conflict monitoring, and the ventrolateral prefrontal cortex is closely associated with cognitive flexibility [4].

This neurobiological mechanism underpinning cognitive control plays a critical role in daily life. During learning, attention control mediated by the dorsolateral prefrontal cortex helps us focus our attention, inhibit distracting information, and thus more effectively absorb and process new knowledge [5]. In work settings, the cognitive flexibility supported by the ventrolateral prefrontal cortex enables us to flexibly switch tasks and adjust strategies, while the conflict monitoring function of the anterior cingulate cortex helps us weigh the pros and cons and make more reasonable judgments [6]. Research indicates that the efficiency of cognitive control supported by specific brain networks is associated with an individual’s academic achievement, occupational performance, and decision-making quality [7,8,9]. These findings not only confirm the neural basis of cognitive control but also provide important biological evidence for understanding individual differences in executive function, while highlighting the core value of cognitive control in human adaptation to complex environments.

In recent years, the impact of exercise on cognitive function has become an important research direction in the fields of exercise science and cognitive neuroscience. Numerous studies have shown that regular exercise not only improves physical health but also enhances cognitive function [10]. In particular, aerobic exercise has been shown to enhance the function of the prefrontal cortex, thereby improving executive function, manifested as faster reaction times and higher task accuracy [11]. However, existing research has notable limitations: most studies focus on acute exercise effects, while research on the long-term impacts of exercise training is relatively scarce [12,13]. Additionally, studies primarily concentrate on closed-domain exercises (such as running and swimming), with limited exploration of open-domain exercises (such as sanda) [14,15].

As a typical open-domain combat sport, sanda offers a unique perspective for studying the relationship between exercise and cognition. Such sports require athletes to process multiple pieces of information simultaneously and make rapid decisions during combat [16]. Preliminary studies indicate that sanda athletes exhibit advantages in specific cognitive tasks, which may be related to the need to continuously address complex situations during training [17]. As an open, combat-oriented sport, sanda requires athletes to constantly endure physical combat and psychological pressure during training and actual combat, which may have a positive impact on cognitive control functions. However, current research on the effects of sanda training on general cognitive functions remains limited, restricting the potential for deeper understanding in this field.

ERP technology offers unique advantages in cognitive neuroscience research, with its core technology based on the application of high-precision EEG sensors. Modern EEG recording systems can non-invasively capture post-synaptic potential activity of cortical neurons at millisecond-level temporal resolution [18]. By using high-density electrode caps and advanced amplifiers, researchers can precisely capture the brain’s electrophysiological responses during different cognitive tasks [19]. These sensor technologies not only enhance the accuracy and reliability of data but also enable ERP studies to explore the neural mechanisms of cognitive control in greater depth. ERP technology has been widely applied in cognitive control research because it can reveal the neural mechanisms underlying cognitive processes, providing detailed information that behavioral indicators cannot reflect [20]. Common ERP components include N200, P300, and N400, which are closely associated with different stages of cognitive control [21].

- N200: A fronto-central negativity peaking 200–350 ms post-stimulus, generated primarily in the anterior cingulate cortex (ACC). Larger N200 amplitudes have been consistently linked to increased conflict monitoring or control recruitment [22].
- P300: A centro-parietal positivity reflecting attentional resource allocation and working-memory updating. Larger P300 amplitudes generally index greater allocation of processing resources to task-relevant information [22].
- N400: A centro-parietal negativity (350–450 ms) sensitive to semantic integration difficulty. Reduced N400 amplitudes are typically interpreted as more efficient semantic processing and suppression of interference [22].

The ERP methodology employed in this study, which relies on high-precision EEG sensors, not only provides insights into the neural mechanisms of cognitive control but also holds promise for applications in wearable or portable EEG platforms. Advances in EEG technology have led to the development of lightweight, wireless systems capable of capturing high-quality neural data in real-world settings. These portable platforms could enable the translation of laboratory-based findings, such as those in this study, to practical scenarios like sports training, cognitive rehabilitation, or performance monitoring. For instance, wearable EEG devices could be used to assess cognitive control in sanda athletes during actual combat or training sessions, providing real-time feedback on neural efficiency and conflict resolution. Future research could explore the feasibility of integrating ERP markers (e.g., N200, P300, N400) into such platforms, offering a non-invasive tool for optimizing cognitive performance in both athletic and clinical populations. This would bridge the gap between experimental neuroscience and everyday applications, further enhancing the impact of ERP-based research.

This study employs event-related potential (ERP) technology to systematically investigate the neural mechanisms underlying the effects of long-term sanda training on individual cognitive control functions. Based on the classic color-word Stroop paradigm, this study focuses on comparing neural electrophysiological differences in conflict adaptation effects between high-level martial arts sanda athletes and ordinary college students. The study hypothesizes that sanda athletes exhibit stronger adaptation effects in conflict-related ERP components, which would provide neurophysiological evidence supporting the notion that long-term sanda training enhances cognitive control abilities. The findings are expected to reveal the neural mechanisms underlying how open-ended movement promotes cognitive function development at the electrophysiological level. This not only holds significant value for refining the theoretical framework linking movement and cognition but also provides new scientific basis for optimizing training protocols and developing cognitive intervention measures.

## 2. Methods

### 2.1. Participants

A total of 38 male participants were recruited from Shanghai University of Sport, comprising two groups:

Sanda Athletes (*n* = 19):

All athletes held national first-level certifications or above (the highest tier in China’s athlete grading system).

Competitive performance: Ranked in the top three in national sanda competitions within the past 3 years.

Training experience: Minimum of 5 years of systematic training (mean ± SD: 7.2 ± 1.5 years).

Training volume: Averaged 15.3 ± 2.1 h per week (including technical drills, sparring, and physical conditioning).

Age: 18–27 years (mean = 23.0 ± 2.3 years).

Ordinary College Students (*n* = 19):

Enrollment status: Full-time undergraduates at Shanghai University of Sport.

Physical activity: No regular exercise habits (≤1 h/week of structured exercise for the past 2 years).

Age: 19–29 years (mean = 23.2 ± 2.1 years).

Inclusion Criteria (Both Groups):

Right-handed (confirmed by the Edinburgh Handedness Inventory).

Normal or corrected-to-normal vision, with no color blindness/weakness (verified by Ishihara tests).

No history of neurological/psychiatric disorders, brain injuries, or musculoskeletal impairments.

No use of psychoactive medications or substances affecting cognition.

Exclusion Criteria:

Participation in other combat sports or high-intensity cognitive training programs.

Recent concussion (within 6 months) or sleep deprivation (<6 h/night before testing).

Abnormal scores on the Beck Depression Inventory (BDI-II > 13) or Generalized Anxiety Disorder Scale (GAD-7 > 9).

All participants provided written informed consent, and the study was approved by the Ethics Committee of Shanghai University of Sport (No. 102772024RT018).

### 2.2. Experimental Design

A 2 × 4 mixed experimental design was employed in this study to investigate the impact of sanda sports experience on cognitive control function. The between-subjects variable was group (sanda athletes and ordinary college students), while the within-subjects variable was four experimental conditions, namely cC (consistent in the previous trial and consistent in the current trial), cI (consistent in the previous trial and inconsistent in the current trial), iC (inconsistent in the previous trial and consistent in the current trial), and iI (inconsistent in the previous trial and inconsistent in the current trial). The experimental task utilized the classic color-word Stroop task, and cognitive control function was assessed by measuring reaction time, accuracy, and ERP amplitude.

### 2.3. Experimental Procedure

The experiment was conducted in a quiet and comfortable shielded room. Participants were seated in a comfortable chair with their head position fixed by a headrest. Before the experiment began, participants completed a practice session consisting of 10 trials to ensure they fully understood the experimental task requirements. During the formal experiment, participants completed two Stroop task sequences with a rest period in between. Participants were required to determine whether the color of the presented word matched its semantic meaning and to press the key “1” with their right index finger to indicate a match and the key “2” with their right middle finger to indicate a mismatch. During the experiment, E-prime 3.0 software was used to present stimuli and record participants’ key-press responses and reaction times. Simultaneously, electroencephalogram (EEG) data were collected using a 64-channel Ag/AgCl electrode cap and a BrainAMP amplifier (manufactured by Brain Products GmbH, Gilching, Germany). The sampling frequency was set at 1000 Hz, with AFz as the ground electrode and FCz as the online reference electrode. The vertical electrooculogram (EOG) was placed about 1 cm below the right eye. Before the start of data collection, the impedance between the electrode points and the scalp was reduced to below 10 kΩ to ensure the accuracy of data collection.

### 2.4. Measurement Indicators

The following indicators were measured in this study:

1. Behavioral Indicators: These included reaction time and accuracy. Reaction time was defined as the time interval from stimulus presentation to participants’ key-press response. Accuracy was defined as the proportion of correctly judged trials out of the total number of trials.
2. ERP Indicators: These included the amplitude of ERP components such as N200, P300, and N400. The time window for N200 was 250–350 ms after stimulus presentation, with electrode sites selected at the mid-frontal region (FPz, Fz, and FCz). The time window for P300 was 300–400 ms after stimulus presentation, with electrode sites selected at the mid-parietal region (Cz, CPz, and Pz). The time window for N400 was 350–450 ms after stimulus presentation, with electrode sites selected at the mid-parietal region (Cz, CPz, and Pz).

### 2.5. Statistical Analysis

#### 2.5.1. Behavioral Data Analysis

Behavioral data were analyzed using SPSS software 22.6. First, the first trial of each sequence was removed, and the overall accuracy and reaction time were calculated. The accuracy and reaction time were then computed for each of the four conditions (cC, cI, iC, and iI). Trials with incorrect responses and reaction times less than 300 ms or greater than the mean plus three standard deviations were excluded. Subsequently, the interference effect (I − C) and conflict adaptation effect [(cI − cC) − (iI − iC)] were calculated.

To control for Type I error inflation due to multiple comparisons across conditions and behavioral measures, the Benjamini–Hochberg false discovery rate (FDR) correction was applied to all ANOVA tests on reaction time and accuracy data. The original significance threshold (*p* < 0.05) was adjusted based on the number of comparisons within each analysis family (e.g., four conditions for reaction time and accuracy, respectively). Only results surviving FDR correction (q < 0.05) were considered statistically significant.

#### 2.5.2. ERP Data Analysis

ERP data were processed using the MATLAB-based EEGLAB (v2021.1) and ERPLAB toolkits V7. The specific steps were as follows: First, the data were re-referenced to the average of the bilateral mastoids. Extended infomax independent component analysis (ICA) was then applied to decompose the data into 30 independent components (equal to the number of scalp electrodes minus rank-deficient channels). Artifact-related components (e.g., eye movements, blinks, muscle activity) were identified through visual inspection of component scalp topographies, time courses, and power spectra, with an average of 3.2 ± 0.8 (mean ± SD) components removed per participant. This manual rejection procedure followed standardized criteria: (1) frontal dipole distribution with high low-frequency power for ocular artifacts, and (2) temporo-parietal localization with high high-frequency power for muscle artifacts. Additionally, epochs exceeding ±100 μV amplitude threshold were automatically rejected.

Subsequently, high-pass filtering was set at 0.1 Hz, and low-pass filtering at 30 Hz. ERP data were time-locked to stimulus presentation (set as zero point), with an analysis time window of −200 ms to 1000 ms. Baseline correction was performed using the period from −200 ms to 0 ms before stimulus presentation.

## 3. Results

### 3.1. Behavioral Results

#### 3.1.1. Reaction Time

As shown in Table 1, the reaction time of the two groups of participants under different experimental conditions was analyzed. The results indicated that the sanda athletes exhibited significantly shorter reaction times than the ordinary college students across all conditions. Specifically, in the cC condition, the reaction time of sanda athletes was significantly shorter than that of ordinary college students (F(1,36) = 9.23, *p* = 0.004). In the cI condition, the reaction time of sanda athletes was also significantly shorter than that of ordinary college students (F(1,36) = 4.21, *p* = 0.048). In the iC condition, the reaction time of sanda athletes was significantly shorter than that of ordinary college students (F(1,36) = 6.79, *p* = 0.012). In the iI condition, the reaction time of sanda athletes was significantly shorter than that of ordinary college students (F(1,36) = 5.95, *p* = 0.020). These results suggest that sanda athletes demonstrated faster response speed in cognitive control tasks.

#### 3.1.2. Accuracy

**Figure 1:**
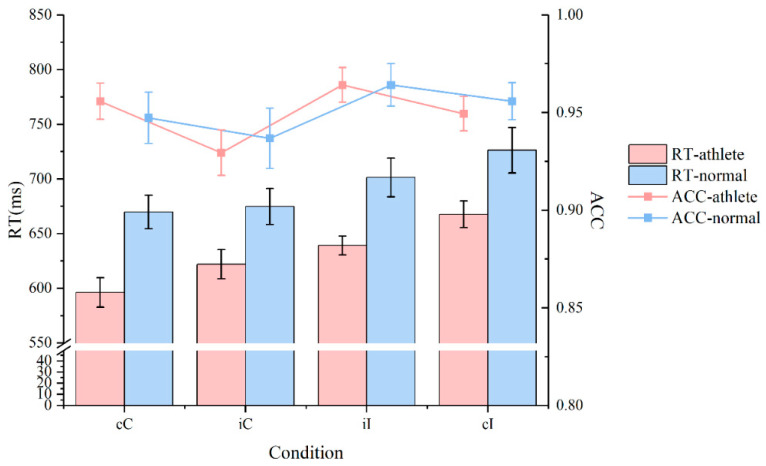
Comparison of behavioral outcomes between athletes and non-athletes across conditions.

Figure 1 and Table 2 presents the accuracy of the two groups of participants under different experimental conditions. The results revealed that there was no significant difference in accuracy between the two groups. Specifically, in the cC condition, there was no significant difference in accuracy between sanda athletes and ordinary college students (F(1,36) = 0.012, *p* = 0.91). In the cI condition, there was no significant difference in accuracy between the two groups (F(1,36) = 0.37, *p* = 0.773). In the iC condition, there was no significant difference in accuracy between the two groups (F(1,36) = 0.61, *p* = 0.44). In the iI condition, there was no significant difference in accuracy between the two groups (F(1,36) = 0.006, *p* = 0.940). These results indicate that sanda athletes did not sacrifice task accuracy while improving response speed.

### 3.2. ERP Results

#### 3.2.1. N200 Component

**Figure 2:**
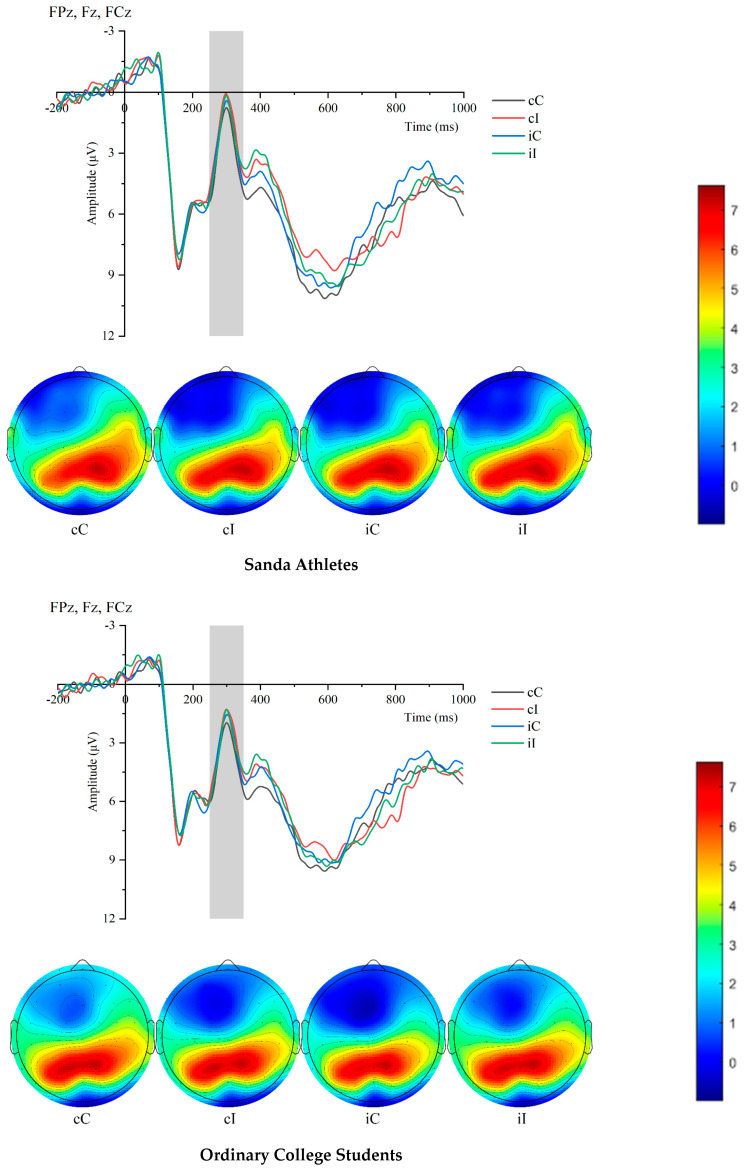
Comparison of N200 waveforms and topographic maps between athletes and non-athletes across conditions.

As shown in Table 3, the N200 amplitude of the two groups of participants under different experimental conditions was analyzed. The results indicated that the sanda athletes exhibited significantly larger N200 amplitude in incongruent trials than in congruent trials. Specifically, in the cC condition, the N200 amplitude of sanda athletes was significantly smaller than that of ordinary college students (F(1,36) = 4.31, *p* = 0.006). In the cI condition, the N200 amplitude of sanda athletes was significantly smaller than that of ordinary college students (F(1,36) = 13.078, *p* < 0.001). In the iC condition, the N200 amplitude of sanda athletes was significantly smaller than that of ordinary college students (F(1,36) = 4.82, *p* = 0.003). In the iI condition, the N200 amplitude of sanda athletes was significantly smaller than that of ordinary college students (F(1,36) = 6.60, *p* = 0.014). Figure 2.

#### 3.2.2. P300 Component

**Figure 3:**
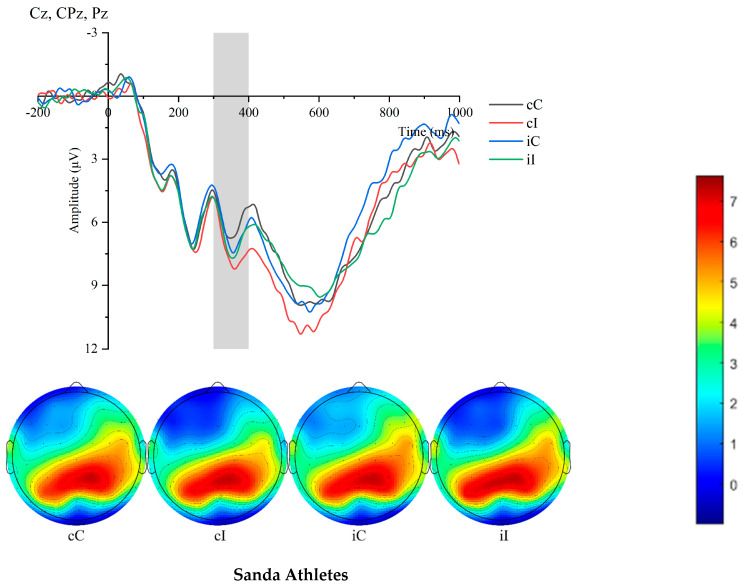

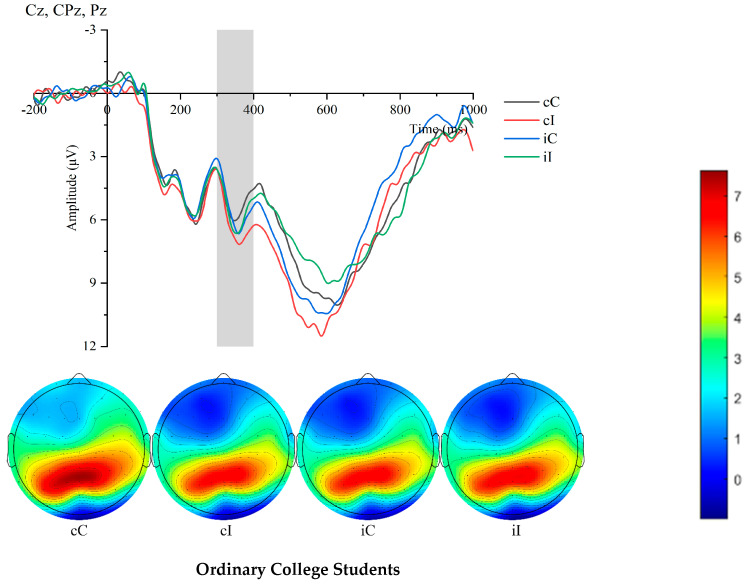
Comparison of P300 waveforms and topographic maps between athletes and non-athletes across conditions.

Table 4 presents the P300 amplitude of the two groups of participants under different experimental conditions. The results revealed that the sanda athletes exhibited significantly larger P300 amplitude in incongruent trials than in congruent trials. Specifically, in the cC condition, the P300 amplitude of sanda athletes was significantly larger than that of ordinary college students (F(1,36) = 4.82, *p* = 0.003). In the cI condition, the P300 amplitude of sanda athletes was significantly larger than that of ordinary college students (F(1,36) = 6.60, *p* = 0.014). In the iC condition, the P300 amplitude of sanda athletes was significantly larger than that of ordinary college students (F(1,36) = 4.31, *p* = 0.006). In the iI condition, the P300 amplitude of sanda athletes was significantly larger than that of ordinary college students (F(1,36) = 13.078, *p* < 0.001). Figure 3.

#### 3.2.3. N400 Component

As shown in Table 5, the N400 amplitude of the two groups of participants under different experimental conditions was analyzed. The results indicated that the sanda athletes exhibited significantly larger N400 amplitude in incongruent trials than the ordinary college students. Specifically, in the cC condition, the N400 amplitude of sanda athletes was significantly larger than that of ordinary college students (F(1,36) = 9.78, *p* < 0.001). In the cI condition, the N400 amplitude of sanda athletes was significantly larger than that of ordinary college students (F(1,36) = 4.289, *p* = 0.046). In the iC condition, the N400 amplitude of sanda athletes was significantly larger than that of ordinary college students (F(1,36) = 3.51, *p* = 0.018). In the iI condition, the N400 amplitude of sanda athletes was significantly larger than that of ordinary college students (F(1,36) = 4.12, *p* = 0.050).

#### 3.2.4. Correlation Analysis Between ERP and Behavior

Athletes

The conflict-adaptation RT index correlated negatively with the N200 amplitude difference (r = −0.538, *p* = 0.017). P300 and N400 differences showed no reliable associations (r = −0.296, *p* = 0.218 and r = −0.441, *p* = 0.059, respectively).

College Students

**Figure 4:**
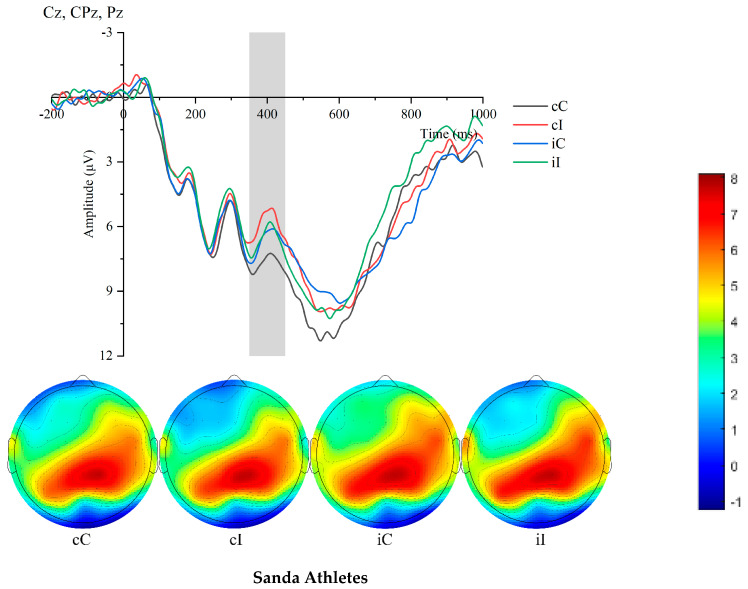

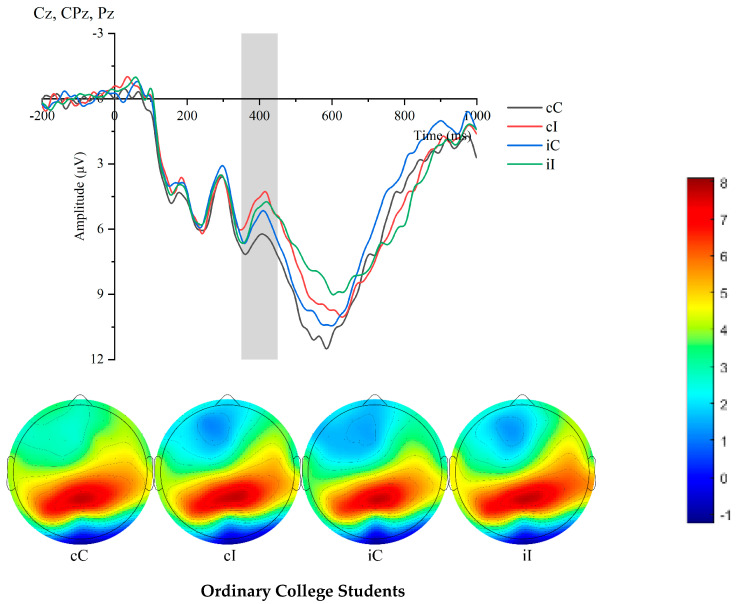
Comparison of N400 waveforms and topographic maps between athletes and non-athletes across conditions.

**Figure 5:**
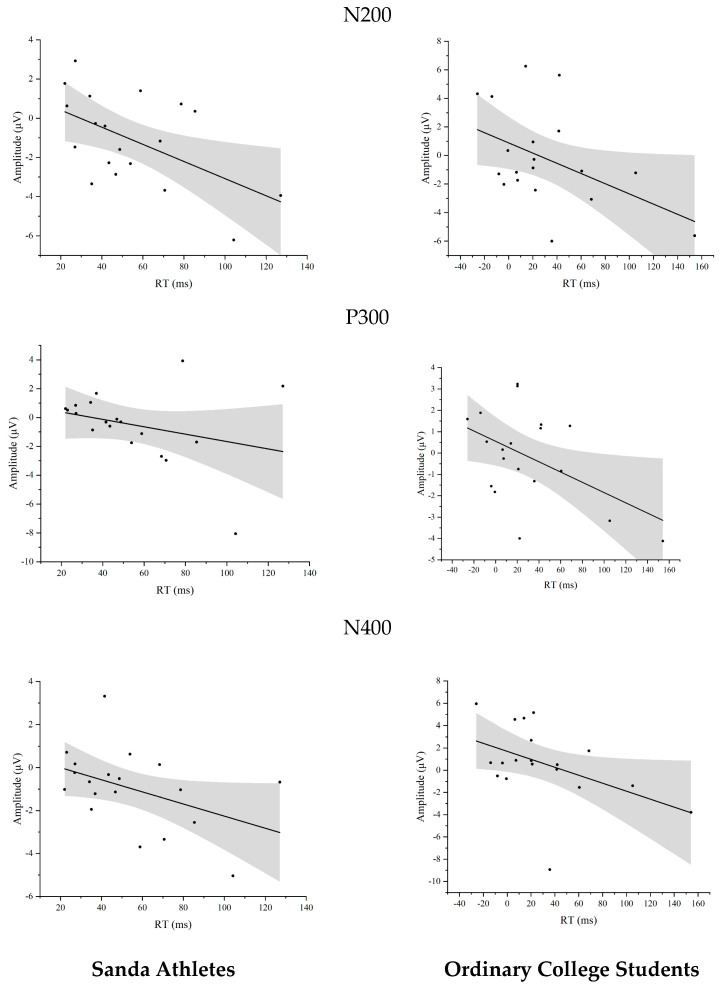
The correlation between wave amplitude and reaction time conflict adaptation effect.

Across all three components, stronger negative ERP differences predicted larger behavioral conflict adaptation: N200 (r = −0.459, *p* = 0.048), P300 (r = −0.487, *p* = 0.034), and N400 (r = −0.457, *p* = 0.049). Figure 4 and Figure 5.

## 4. Discussion

### 4.1. Behavioral

The behavioral results of this study showed that the reaction times of sanda athletes were significantly shorter than those of ordinary college students under all experimental conditions, but there was no significant difference in task accuracy between the two groups. This finding is consistent with previous studies on the cognitive control advantages of athletes, suggesting that long-term specialized training may optimize behavioral performance by enhancing information processing speed [23]. However, it is important to note that the cross-sectional design of this study cannot rule out the possibility that pre-existing differences in cognitive abilities may have influenced these results. Individuals with naturally faster processing speeds might self-select into sanda training, or genetic factors could contribute to both athletic performance and cognitive efficiency.

Nevertheless, the observed balance between speed and accuracy in athletes aligns with the dual demands of sanda for rapid decision-making and precise movement control [24]. Sanda training requires athletes to simultaneously perform attacks, defenses, and tactical adjustments in high-speed combat, and this multitasking experience may promote the flexible allocation of cognitive resources. Future longitudinal studies tracking cognitive changes in novice athletes before and after training, or randomized controlled trials assigning participants to sanda versus control interventions, would help clarify whether these effects are directly attributable to training.

Additionally, this study did not observe differences in accuracy between the two groups under conflict conditions, which contrasts with some studies on open-sport athletes [25]. This discrepancy may stem from the specificity of the experimental task: this study employed a classical conflict paradigm, whereas conflicts in sanda are more manifested as dynamic physical interactions, whose neural mechanisms may involve more complex perception-action coupling systems. Future studies could further validate this by incorporating sport-specific tasks.

### 4.2. ERP

The N200 amplitude of sanda athletes was significantly smaller than that of ordinary college students under conflict conditions. While larger N200 amplitudes are traditionally associated with enhanced conflict monitoring and cognitive control in the anterior cingulate cortex (ACC) [26], the observed reduction in athletes may reflect a more efficient neural response due to long-term training. Specifically, sanda athletes’ extensive experience in rapidly resolving physical and tactical conflicts could lead to a streamlined neural mechanism for conflict detection, requiring less activation for equivalent performance. This interpretation aligns with the “Neural Efficiency Hypothesis”, where experts exhibit optimized neural resource allocation [27]. However, future studies should investigate whether this reflects training-induced plasticity or pre-existing neural efficiency.

The smaller N200 amplitude in athletes might also indicate faster conflict resolution, as ACC activity in experts could peak earlier or involve fewer resources due to automated inhibitory processes [27]. This is consistent with sanda’s demands for rapid discrimination between relevant and irrelevant stimuli during combat.

Athletes exhibited significantly larger P300 amplitudes across all conditions, with more pronounced differences in conflict tasks. The P300 reflects attentional resource allocation and working memory updating [28], and its enhancement in athletes supports the idea that sanda training strengthens the ability to mobilize cognitive resources under demanding conditions. Larger P300 amplitudes are typically linked to greater engagement of task-relevant neural networks, suggesting that athletes allocate more resources to maintain high performance during conflict tasks. This aligns with the “Neural Compensation Hypothesis”, where experts may recruit additional resources to achieve superior performance [28].

Notably, the P300 advantage of sanda athletes was more pronounced under incongruent conditions, which may reflect their heightened sensitivity to unexpected stimuli due to training in unpredictable combat scenarios.

Athletes exhibited larger N400 amplitudes, particularly under semantic conflict conditions. While reduced N400 amplitudes are traditionally associated with more efficient semantic processing [29], the increased amplitude in athletes could reflect deeper semantic analysis or heightened sensitivity to interference. In the context of sanda, where rapid interpretation of tactical language (e.g., coach instructions) and situational judgment are critical, larger N400 amplitudes may indicate enhanced semantic monitoring or prolonged conflict resolution. This could represent a sport-specific adaptation, where athletes engage in more thorough evaluation of conflicting semantic information [30].

Alternatively, the larger N400 in athletes might reflect greater neural effort to suppress irrelevant semantic interference, as sanda training emphasizes filtering distractions during high-stakes decision-making. Future studies should clarify whether this pattern reflects adaptive plasticity or task-specific demands.

### 4.3. Limitations

While this study provides novel neurophysiological evidence for sanda’s cognitive benefits, three key limitations should be noted. First, the cross-sectional design cannot establish causality between training and observed effects; future longitudinal studies tracking novices through training progression, or randomized controlled trials assigning participants to sanda versus control interventions, are needed. Second, the Stroop task, while validated for cognitive control assessment, lacks sport-specific ecological validity. Future research should incorporate dynamic paradigms mimicking sanda’s perceptual-motor demands, such as virtual reality combat scenarios with simultaneous EEG monitoring. Third, the male-only sample limits generalizability—expanding to female athletes and varied skill levels would strengthen conclusions. Despite these constraints, our findings establish foundational evidence for sanda-induced neuroplasticity.

## 5. Conclusions

This study demonstrates that long-term sanda training enhances cognitive control, as evidenced by faster reaction times and improved neural efficiency in conflict monitoring (N200), cognitive resource allocation (P300), and interference suppression (N400) compared to non-athletes. These findings suggest that open-domain combat sports like sanda promote neuroplasticity in executive function networks, optimizing both behavioral performance and neural processing. Future research should explore causal mechanisms through longitudinal designs and more ecologically valid tasks.

## Figures and Tables

**Table 1:** Reaction Time of the Two Groups of Participants Under Different Conditions (Unit: Milliseconds).

Experimental Condition	Sanda Athletes (*n* = 19)	Ordinary College Students (*n* = 19)	F-Value	*p*-Value	η^2^
cC	596.21 ± 58.59	669.80 ± 67.44	9.23	0.004	0.204
cI	667.72 ± 53.34	726.31 ± 90.42	4.21	0.048	0.105
iC	622.13 ± 57.97	674.81 ± 71.85	6.79	0.012	0.159
iI	639.26 ± 37.26	701.46 ± 77.07	5.95	0.020	0.142

Note: Data are presented as mean ± standard deviation.

**Table 2:** Accuracy of the Two Groups of Participants Under Different Conditions.

Experimental Condition	Sanda Athletes (*n* = 19)	Ordinary College Students (*n* = 19)	F-Value	*p*-Value	η^2^
cC	0.956 ± 0.04	0.947 ± 0.057	0.012	0.91	0.000
cI	0.949 ± 0.039	0.956 ± 0.041	0.37	0.773	0.010
iC	0.929 ± 0.051	0.937 ± 0.067	0.61	0.44	0.016
iI	0.964 ± 0.039	0.964 ± 0.047	0.006	0.940	0.000

Note: Data are presented as mean ± standard deviation.

**Table 3:** N200 Amplitude of the Two Groups of Participants Under Different Conditions (Unit: Microvolts).

Experimental Condition	Sanda Athletes (*n* = 19)	Ordinary College Students (*n* = 19)	F-Value	*p*-Value	η^2^
cC	2.845 ± 4.534	3.750 ± 4.688	4.31	0.006	0.107
cI	2.070 ± 4.004	3.057 ± 4.120	13.078	<0.001	0.267
iC	2.400 ± 4.775	3.321 ± 4.573	4.82	0.003	0.118
iI	2.379 ± 4.222	3.312 ± 4.139	6.60	0.014	0.155

Note: Data are presented as mean ± standard deviation.

**Table 4:** P300 Amplitude of the Two Groups of Participants Under Different Conditions (Unit: Microvolts).

Experimental Condition	Sanda Athletes (*n* = 19)	Ordinary College Students (*n* = 19)	F-Value	*p*-Value	η^2^
cC	5.945 ± 3.500	5.161 ± 3.434	4.82	0.003	0.118
cI	7.191 ± 4.555	6.122 ± 4.062	6.60	0.014	0.155
iC	6.315 ± 4.083	5.404 ± 4.146	4.31	0.006	0.107
iI	6.641 ± 4.003	5.483 ± 3.637	13.078	<0.001	0.267

Note: Data are presented as mean ± standard deviation.

**Table 5:** N400 Amplitude of the Two Groups of Participants Under Different Conditions (Unit: Microvolts).

Experimental Condition	Sanda Athletes (*n* = 19)	Ordinary College Students (*n* = 19)	F-Value	*p*-Value	η^2^
cC	7.718 ± 4.773	6.670 ± 4.709	9.78	<0.001	0.214
cI	5.883 ± 4.050	5.002 ± 4.228	4.289	0.046	0.106
iC	6.683 ± 4.400	5.421 ± 4.163	3.51	0.018	0.089
iI	6.603 ± 4.402	5.864 ± 4.537	4.12	0.050	0.103

Note: Data are presented as mean ± standard deviation.

## Data Availability

Data are contained within the article.
